# Demographic Bottlenecks and Size-Dependent Competition Shape *Populus euphratica* Persistence

**DOI:** 10.3390/plants15172662

**Published:** 2026-08-30

**Authors:** Yuhuang Lin, Qiao Li, Ying Yang, Chunxia Wei, Ziwei Dou, Qingchun Wang

**Affiliations:** 1 School of Ecology and Nature Conservation, Beijing Forestry University, Beijing 100083, China; yhlin13@163.com (Y.L.); yying47@163.com (Y.Y.); 2 Xinjiang Tarim Poplar National Nature Reserve Administration, Korla 841000, China

**Keywords:** diameter-class structure, Hegyi competition index, local neighbourhood density, competitive filtering, Tarim River Basin, arid riparian forest

## Abstract

Although *Populus euphratica* forests are key components of arid riparian ecosystems, the demographic and competitive processes associated with their population persistence remain insufficiently understood. We investigated population structure, demographic dynamics and intraspecific competition in a natural *P. euphratica* population within the Tarim River Basin, northwestern China. Field data from 2690 individuals were analyzed using diameter-class structure, population dynamic indices and the Hegyi competition index. The population exhibited an inverse J-shaped diameter distribution: Class I contained 1601 individuals (59.5% of the total population), and Classes I–III together accounted for 83.9%, indicating active recruitment but limited transition into intermediate diameter classes. Competition intensity was negatively correlated with DBH, tree height and crown area (r = −0.42, −0.41 and −0.31, respectively; *p* < 0.001) but positively correlated with local neighbourhood density (r = 0.39; *p* < 0.001). The multiple regression model explained 27.1% of the variation in competition intensity, and the DBH power-law model provided the lowest residual standard error (RSE = 4.887). These findings suggest that demographic bottlenecks and size-dependent competition are closely associated with *P. euphratica* persistence and provide field-based evidence for competitive filtering in arid riparian tree populations.

## 1. Introduction

Riparian forest ecosystems play an irreplaceable role in maintaining ecological stability, biodiversity conservation, soil protection and hydrological regulation in arid regions. Among these ecosystems, *Populus euphratica* is the dominant constructive tree species distributed along inland river systems throughout Central Asia and northwestern China [1,2]. In the Tarim *Populus euphratica* National Nature Reserve, *P. euphratica* forms the main woody canopy component of the desert riparian forest, together with Tamarix spp. and other shrubs in the understory. Owing to its remarkable tolerance to drought, salinity and temperature extremes, *P. euphratica* provides essential ecosystem services, including sand fixation, carbon sequestration and habitat maintenance [3,4,5]. These ecological functions also explain why this species is a priority target for conservation and monitoring in arid inland river systems.

Over recent decades, however, increasing environmental pressures associated with climate change, river regulation, groundwater decline and anthropogenic disturbance have substantially altered the structure and functioning of *P. euphratica* forests [6,7,8,9,10,11]. Numerous studies have reported widespread forest degradation, reduced regeneration capacity, increased mortality of mature trees and fragmentation of forest patches throughout the Tarim River Basin [12,13,14,15]. These changes have raised considerable concern regarding the long-term persistence and sustainability of desert riparian forests [16,17].

Population structure represents one of the most important indicators for evaluating the developmental status and regeneration potential of forest ecosystems [15,18]. Because direct age determination is often difficult in natural forests, diameter-class structure is commonly used as a surrogate for age structure [15,19,20]. Diameter distributions provide valuable information regarding population recruitment, survival and growth processes, thereby enabling inference of future developmental trajectories. Previous studies have demonstrated that *P. euphratica* populations may exhibit inverse J-shaped, unimodal or declining diameter distributions depending on hydrological conditions, habitat quality and disturbance regimes [21,22].

Although population structure provides important information regarding current demographic status, it cannot independently explain the mechanisms underlying population persistence and succession. Similar diameter distributions may result from fundamentally different demographic processes. Therefore, understanding population development requires integration of structural patterns with population dynamics.

Population dynamics constitute the critical link between demographic structure and ecological processes [15,23,24]. The transition efficiency among successive size classes directly determines the regeneration capacity and long-term maintenance of plant populations. Here, transition efficiency refers to the relative continuity of individuals among successive diameter classes, reflecting whether recruited individuals can progress into intermediate and larger size classes. In water-limited ecosystems, population dynamics frequently exhibit strong stage dependence, with active recruitment at early developmental stages but reduced survival and growth during later stages [25,26]. Such unequal transitions among size classes are widely recognized as a characteristic feature of forest populations in arid environments. However, the ecological mechanisms driving these patterns remain incompletely understood.

Intraspecific competition represents another fundamental process regulating population development [27]. Competition intensity is jointly influenced by individual size, spatial arrangement and local resource availability. Small individuals generally experience stronger suppression because of their relatively weak competitive ability, whereas larger trees tend to dominate resource acquisition [28]. At the same time, spatial aggregation may substantially increase local competition intensity, leading to unequal resource distribution among neighborhood individuals. Consequently, competitive interactions often exhibit pronounced scale-dependent and density-dependent characteristics [29,30].

Previous investigations of *P. euphratica* forests have primarily focused on spatial distribution patterns, regeneration characteristics or competitive interactions independently [20,31]. These studies have substantially advanced understanding of population ecology in desert riparian forests. Nevertheless, important limitations remain. First, most studies have examined population structure, dynamics or competition separately, lacking an integrated framework capable of linking these processes. Second, comparative analyses among different habitat conditions remain limited, constraining our understanding of the mechanisms responsible for spatial variation in population development.

This gap is particularly important in resource-limited ecosystems, where recruitment alone may not ensure long-term population persistence. Population persistence depends not only on recruitment but also on the successful transition of individuals through successive developmental stages. High recruitment may produce an apparent inverse J-shaped structure, but strong intraspecific competition can reduce growth and survival during later stages, generating demographic bottlenecks. Therefore, linking population structure with demographic transitions and competition intensity is essential for understanding how dominant plant populations persist under environmental stress.

Population structure, demographic dynamics and competitive interactions should not be viewed as isolated phenomena [22,30]. Rather, they represent interconnected components of a unified population regulation system. Structural characteristics influence competitive environments, and competition affects individual growth and survival; these processes ultimately determine population dynamics and future structural development. Therefore, integrating these components within a common analytical framework is essential for understanding the mechanisms governing population persistence in arid riparian forests [32].

In this study, we examined three related hypotheses: (1) *P. euphratica* populations exhibit an inverse J-shaped diameter structure, but transitions into intermediate diameter classes are constrained; (2) competition intensity decreases with tree size but increases with local neighborhood density; and (3) size-dependent competition is associated with demographic filtering during stage-dependent population development. By examining these hypotheses, this study aims to clarify how demographic bottlenecks and competition are associated with *P. euphratica* persistence in arid riparian ecosystems.

## 2. Results

### 2.1. Population Structure

#### 2.1.1. Overall Diameter-Class Structure

**Figure 1:**
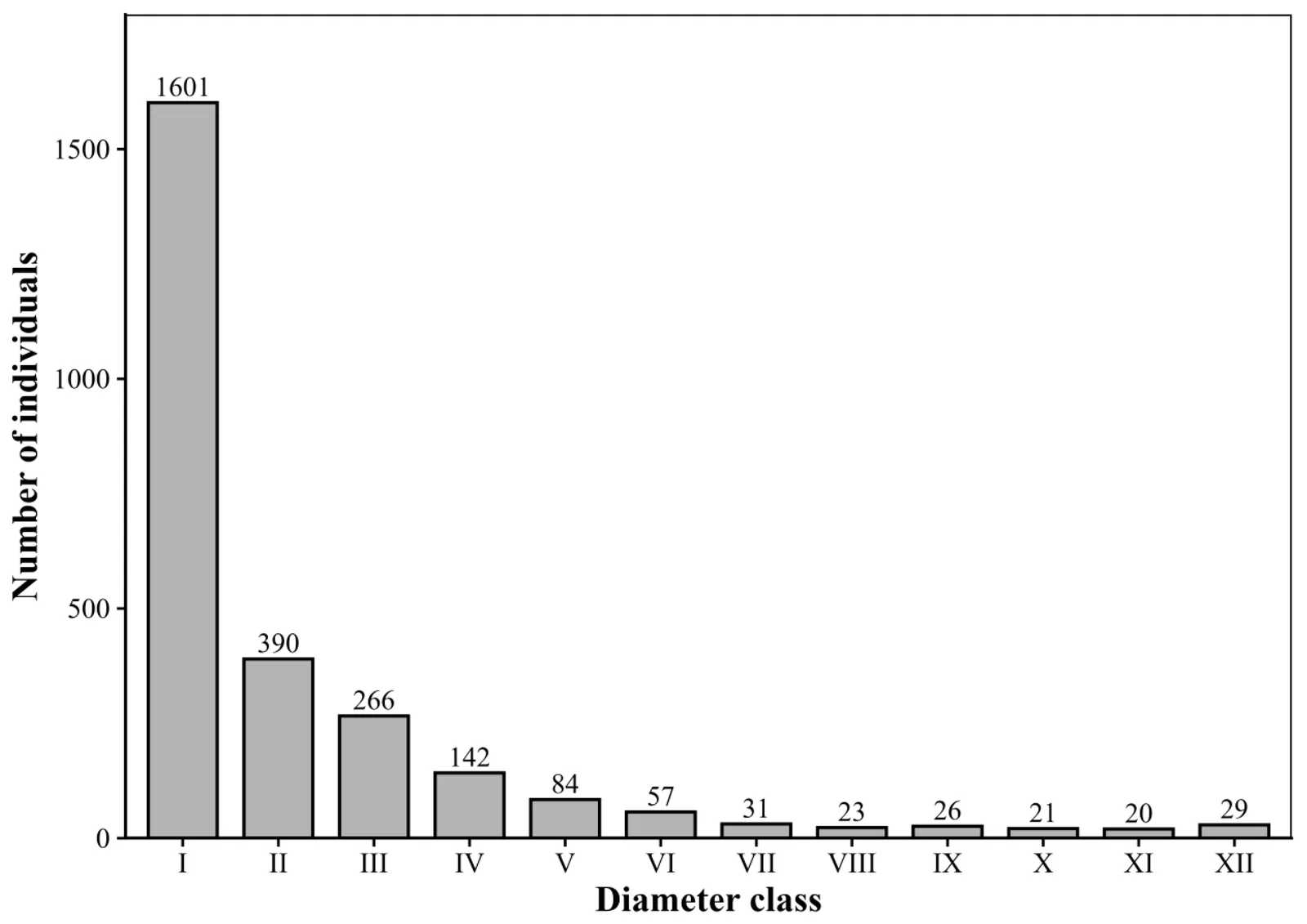
Diameter-class structure of the overall population. Diameter classes were defined at 4 cm DBH intervals: Class I, DBH < 4 cm; Class II, 4 ≤ DBH < 8 cm; …; Class XII, DBH ≥ 44 cm.

The overall diameter-class distribution of *Populus euphratica* exhibited a typical inverse J-shaped pattern (Figure 1). The abundance of individuals was highest in the smallest diameter class (Class I) and progressively decreased with increasing diameter class. Although slight fluctuations occurred in several intermediate classes, the overall declining trend remained evident throughout the population.

The dominance of small-diameter individuals indicates continuous recruitment and strong regeneration capacity within the study area. Individuals belonging to Classes I–III accounted for the majority of the population, indicating a high abundance of small-diameter individuals. In contrast, the abundance of individuals in Classes VIII–XII was relatively low, suggesting that only a small proportion of individuals successfully reached mature developmental stages.

A pronounced reduction in abundance was observed in several intermediate diameter classes. The abundance declined sharply from small to intermediate diameter classes, indicating reduced representation of individuals in intermediate classes. Consequently, although the population currently possesses considerable regeneration potential, the accumulation of mature individuals remains limited.

Overall, the population showed active recruitment but reduced representation of individuals in intermediate and large diameter classes.

#### 2.1.2. Plot-Level Comparisons

**Figure 2:**
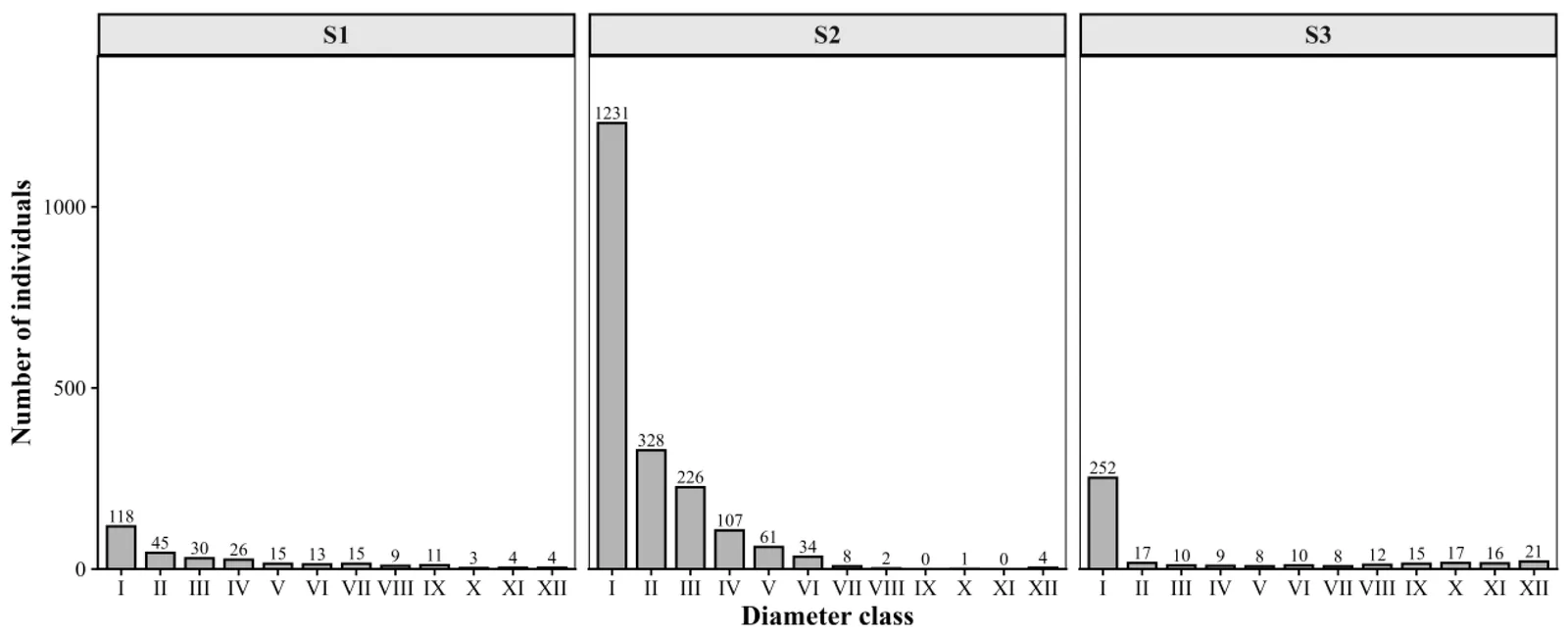
Diameter-class distributions of *Populus euphratica* populations in plots S1, S2 and S3. Diameter classes were defined at 4 cm DBH intervals from Class I to Class XII.

Substantial differences in diameter-class structure were observed among plots (Figure 2).

Plot S1 exhibited the strongest inverse J-shaped pattern, with a large proportion of individuals concentrated in the smallest diameter classes. The continuous decline in abundance across diameter classes indicates active regeneration and a relatively stable developmental trajectory.

Plot S2 also displayed an inverse J-shaped distribution; however, fluctuations among intermediate classes were more pronounced, indicating uneven representation among developmental stages.

Compared with S1 and S2, Plot S3 exhibited a relatively flatter diameter distribution, with a greater representation of intermediate and large individuals, suggesting weaker dominance of newly recruited individuals.

The overall population structure represents the combined effects of these contrasting demographic patterns. Differences among plots indicate spatial heterogeneity in diameter-class structure and population development.

### 2.2. Population Dynamics

#### 2.2.1. Dynamic Characteristics Among Diameter Classes

**Figure 3:**
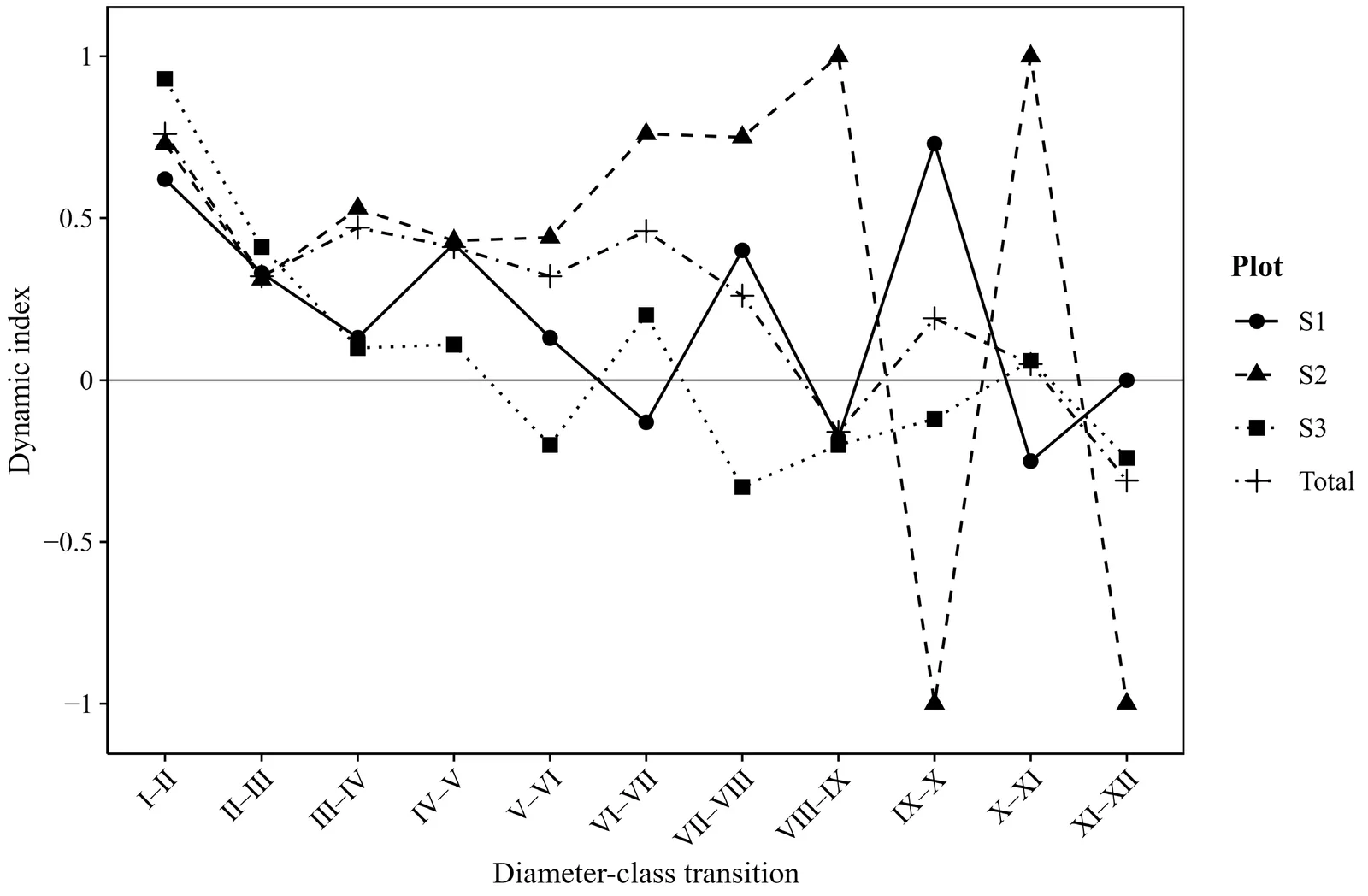
Dynamic indices between adjacent diameter classes of *Populus euphratica* populations in plots S1, S2, S3 and the total population. V1–V11 represent transitions from Class I–II to Class XI–XII.

Dynamic indices calculated between adjacent diameter classes revealed considerable variation across developmental stages (Table 1 and Figure 3).

V_1_–V_11_ represent transitions between adjacent diameter classes from Class I–II to Class XI–XII. The indices are dimensionless ratios.

Positive dynamic values were primarily observed among early diameter classes, indicating greater abundance in smaller classes than in subsequent classes. In contrast, transitions among intermediate diameter classes frequently exhibited lower values, indicating reduced representation of individuals in these classes.

The strongest demographic fluctuations generally occurred in the transition from juvenile to intermediate stages. Transitions involving intermediate diameter classes showed lower or negative dynamic values, indicating reduced representation of individuals in these classes.

Several diameter-class transitions exhibited negative dynamic values, indicating declines in abundance between successive classes. Such declines indicate reduced representation of individuals between successive diameter classes.

Overall, population dynamics were characterized by rapid recruitment in early stages and progressively reduced transition rates toward larger diameter classes.

#### 2.2.2. Population Stability Analysis

The corrected dynamic index (V_pi_′) and maximum dynamic index (P_max_) further revealed differences in population stability among plots (Table 2).

At the population level, the overall dynamic index (V_pi_) indicated active population development, whereas the corrected dynamic index (V_pi_′) and P_max_ suggested that demographic stability differed among plots and remained sensitive to the abundance of the least represented diameter classes.

P_max_ values demonstrated that population stability varied considerably among plots. Lower P_max_ values indicate lower maximum sensitivity to demographic fluctuation, whereas higher values indicate greater developmental uncertainty.

These results indicate that transitions through intermediate diameter classes represented an important source of variation in population stability.

#### 2.2.3. Comparison Among Plots

Among the three plots, S2 exhibited the strongest demographic fluctuation among adjacent diameter classes, whereas S3 showed a relatively flatter diameter-class distribution and weaker dominance of small individuals. S1 showed comparatively stable demographic transitions.

Overall, differences in V_n_, V_pi_′ and P_max_ indicate spatial variation in population dynamics within the reserve.

### 2.3. Competition Characteristics

#### 2.3.1. Correlation Analysis

**Figure 4:**
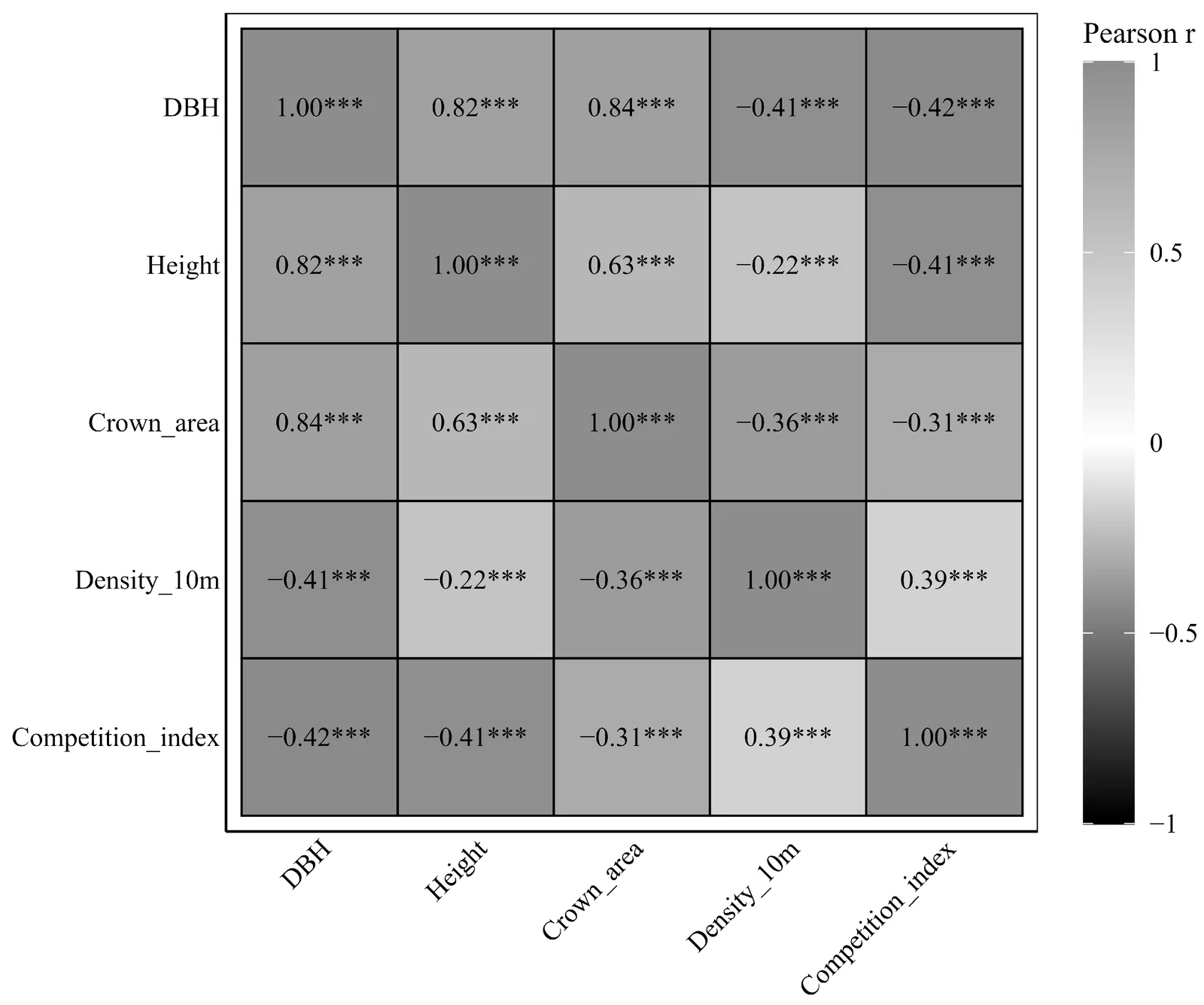
Pearson correlation heat-map. Asterisks indicate statistical significance: *** *p* < 0.001.

Pearson correlation analysis revealed significant relationships among competition intensity, tree size attributes and stand structure (Figure 4).

Competition index was negatively correlated with DBH, tree height and crown area, indicating that larger individuals generally experienced lower competitive pressure. In contrast, competition intensity increased significantly with local neighborhood density.

Strong correlations were also observed among DBH, height and crown area, reflecting coordinated growth patterns among structural attributes. A scatterplot matrix showing the pairwise relationships among variables has been provided as Supplementary Figure S1.

These results indicate that competition intensity was associated with both individual size and local crowding.

#### 2.3.2. Linear Regression Analysis

**Figure 5:**
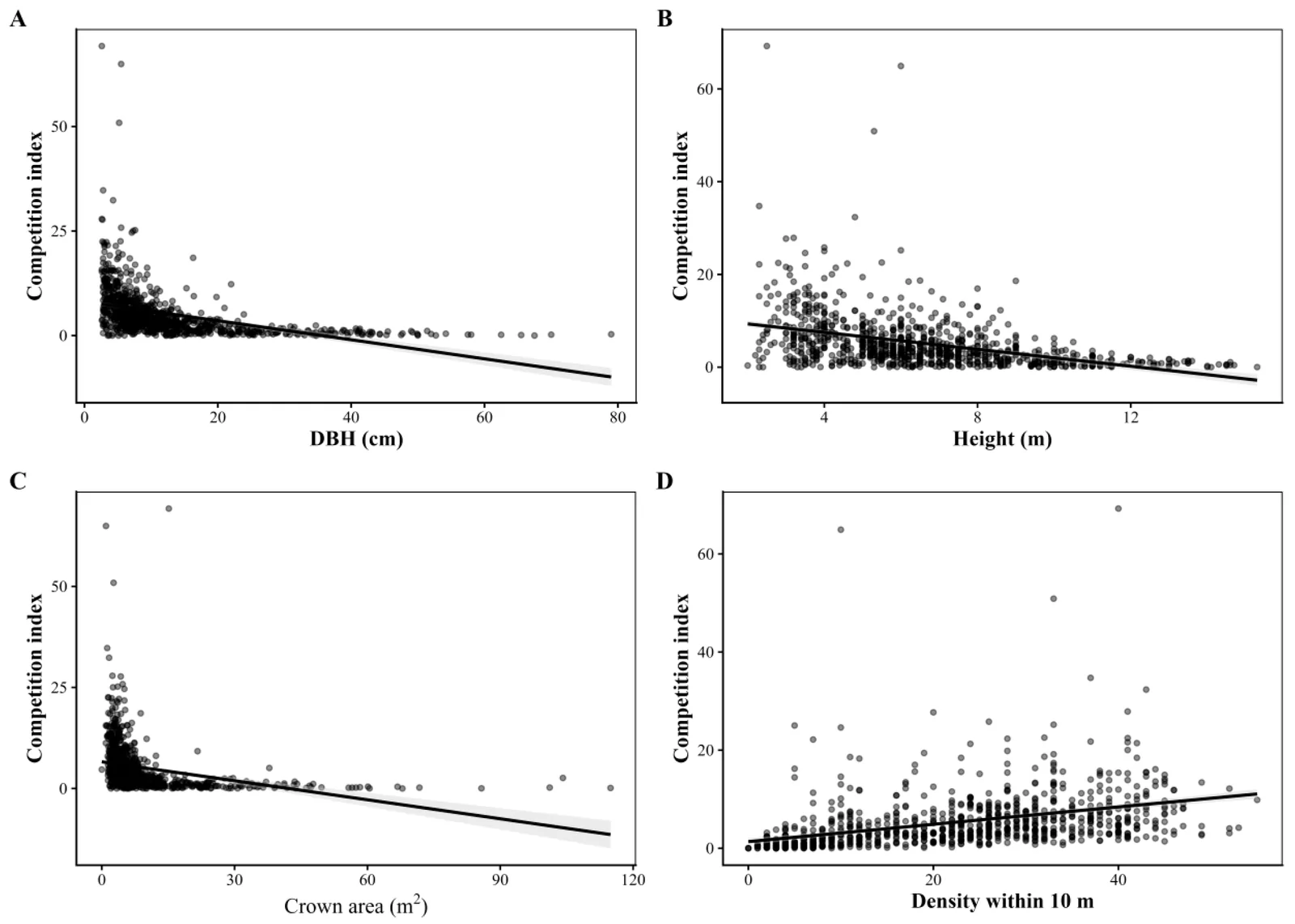
Single-factor linear regression models relating competition intensity and explanatory variables: (**A**) DBH; (**B**) tree height; (**C**) crown area; and (**D**) local neighborhood density. Figure 5. Single-factor linear regression models relating competition intensity to explanatory variables: (**A**) DBH; (**B**) tree height; (**C**) crown area; and (**D**) local neighborhood density. Points represent individual trees, solid lines indicate fitted linear regression relationships, and shaded bands indicate 95% confidence intervals.

Simple linear regression analyses showed that competition intensity was significantly related to all four explanatory variables. Competition intensity decreased significantly with increasing DBH, tree height and crown area but increased significantly with local neighborhood density. These single-factor linear relationships are shown in Figure 5 and summarized in Table 3.

Among the single-factor models, DBH explained the largest proportion of variation in competition intensity, followed by tree height and local neighborhood density.

#### 2.3.3. Multiple Regression Analysis

Multiple linear regression further showed that DBH, tree height, crown area and local neighborhood density jointly explained variation in competition intensity (Table 4).

Local neighborhood density had the strongest positive effect, whereas tree height had the strongest negative effect. Variance inflation factors (VIFs) ranged from 1.27 to 7.16, indicating that multicollinearity was within an acceptable range. The positive coefficient of crown area should therefore be interpreted as a conditional effect after controlling for other size-related variables, rather than as a simple bivariate relationship.

#### 2.3.4. Nonlinear Model Fitting

**Figure 6:**
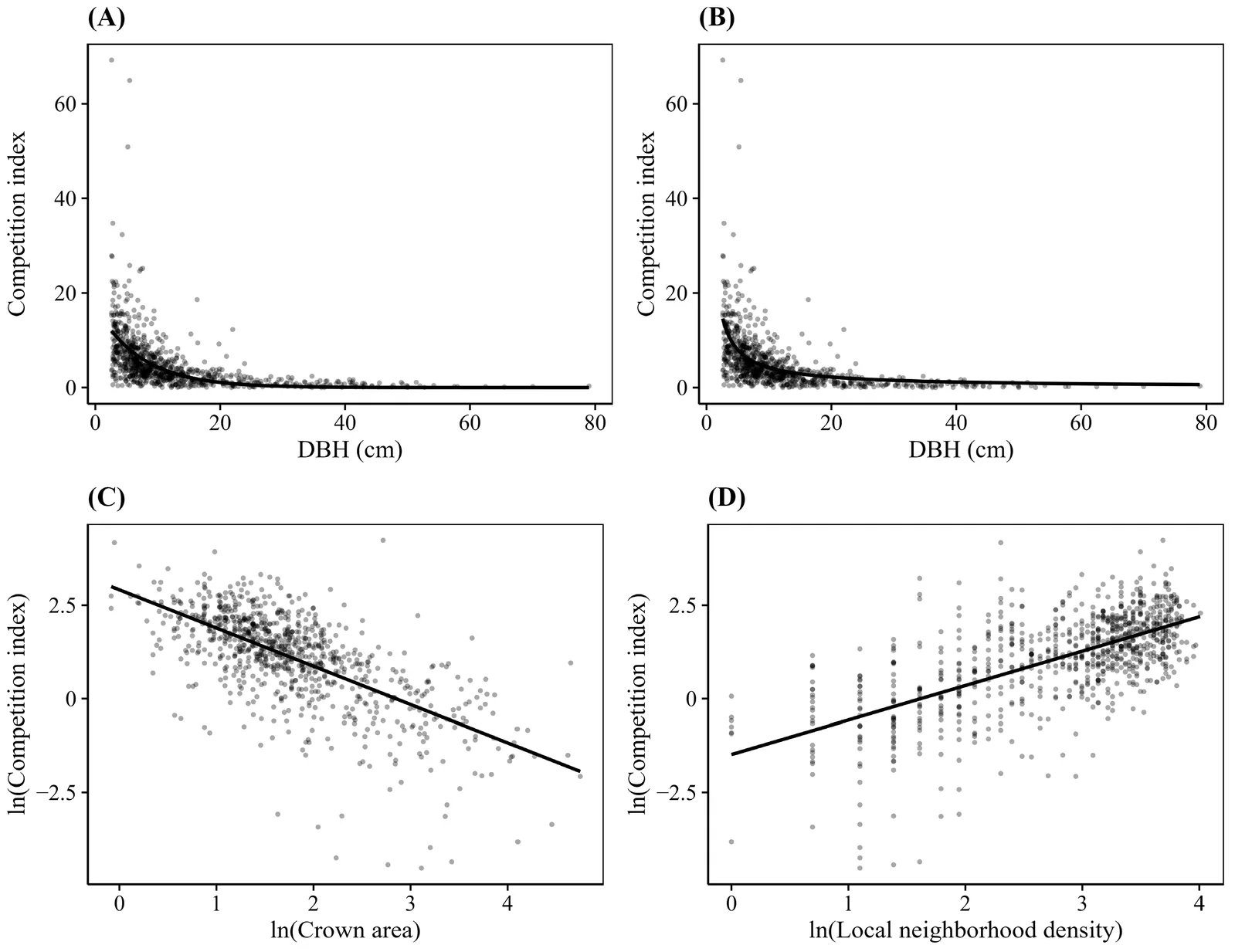
Nonlinear and log-transformed models of competition intensity: (**A**) exponential model relating DBH and competition index; (**B**) power-law model relating DBH and competition index; (**C**) log-transformed model relating crown area and competition index; and (**D**) log-transformed model relating local neighborhood density and competition index. Points represent individual trees, and solid lines indicate fitted model relationships.

Nonlinear regression models provided improved descriptions of competition patterns. The fitted nonlinear and log-transformed relationships are shown in Figure 6 and summarized in Table 5.

These results indicate that competition intensity responded nonlinearly to variations in tree size and local neighborhood density, with a rapid decline in competition intensity as DBH increased.

#### 2.3.5. Integrated Patterns of Population Structure and Competition

Active recruitment, reduced representation of intermediate diameter classes and higher competition among smaller individuals occurred simultaneously. Local neighborhood density was positively associated with competition intensity. These results provide the empirical basis for linking population structure, stage-dependent transitions and neighborhood competition in the Discussion.

## 3. Discussion

### 3.1. Population Structure and Demographic Bottlenecks

The first hypothesis was supported by the observed inverse J-shaped diameter distribution and by the sharp decline from small to intermediate diameter classes. Across the full dataset, Class I contained 1601 individuals, accounting for 59.5% of the total population, whereas Classes I–III together accounted for 83.9%. These values indicate active recruitment and a large pool of juvenile individuals. Similar diameter-class approaches have been widely used to infer regeneration status and demographic development in long-lived woody plant populations [15,19,20,23]. However, the much lower abundance of individuals belonging to the intermediate and large diameter classes shows that recruitment alone does not guarantee continuous demographic progression.

The dynamic indices further confirmed that population development was stage-dependent. Positive values were mainly concentrated in early transitions, whereas several intermediate transitions showed low or negative values. This pattern indicates that intermediate diameter classes represent a demographic bottleneck. Such bottlenecks should not be interpreted simply as a detrimental process or as management failure; rather, they reflect stage-dependent demographic regulation in which only a subset of recruited individuals successfully progress to later developmental stages. Comparable regeneration constraints and stage-dependent mortality patterns have also been reported in Tarim River *P. euphratica* forests under altered hydrological conditions [24,33].

These findings refine the interpretation of inverse J-shaped structures in long-lived riparian trees. Although such structures are commonly interpreted as evidence of regeneration, they may conceal restricted progression through intermediate stages. Therefore, the demographic status of *P. euphratica* populations should be evaluated not only by the abundance of small individuals but also by the continuity of transitions into intermediate and larger diameter classes.

### 3.2. Tree Size, Local Neighborhood Density and Competition Intensity

The second hypothesis was also supported. Competition intensity decreased with increasing DBH, tree height and crown area but increased with local neighborhood density. Pearson correlation coefficients showed negative associations between competition intensity and DBH, height and crown area (r = −0.42, −0.41 and −0.31, respectively; *p* < 0.001), whereas the correlation with local neighborhood density was positive (r = 0.39; *p* < 0.001). These numerical results indicate that competition in *P. euphratica* populations is both size-dependent and density-dependent. This pattern is consistent with plant competition theory, which emphasizes that competitive effects depend on individual size, local density and spatial arrangement [27,28,29,30].

The multiple regression model explained 27.1% of the variation in competition intensity. Local neighborhood density had the strongest positive coefficient, while tree height had the strongest negative coefficient. This result suggests that crowding by neighboring individuals increases competitive pressure, whereas larger trees generally experience lower competition. The VIF values ranged from 1.27 to 7.16, indicating that multicollinearity was acceptable and that the model could be interpreted cautiously.

The nonlinear models further showed that competition intensity declined rapidly with tree size. The DBH power-law model had the lowest residual standard error (RSE = 4.887), indicating that the decline in competition was not purely linear. This pattern is consistent with size-asymmetric competition and allometric size-hierarchy theory, in which relatively small changes in early tree size may substantially alter resource acquisition and competitive status. Power-law responses are also consistent with allometric theory linking plant size, resource acquisition and population density relationships [34,35].

### 3.3. Competition, Demographic Filtering and Population Persistence

The results do not directly measure individual survival, growth or transition probabilities, but they show that reduced representation of intermediate diameter classes occurred together with stronger competition among smaller individuals and higher competition in crowded neighborhoods. These patterns support the interpretation that size-dependent competition is associated with demographic filtering during stage-dependent population development. This interpretation is consistent with demographic theory showing that stage-specific transitions strongly affect plant population growth and with neighborhood competition studies showing that local crowding can influence individual performance [23,36,37].

This pattern is consistent with the possibility that local crowding and size-asymmetric competition may limit the progression of some individuals into intermediate and mature stages. The same demographic pattern that generates an inverse J-shaped structure may therefore be associated with competition-sensitive bottlenecks if many juveniles remain suppressed or fail to progress. This interpretation helps explain why high recruitment does not necessarily translate into long-term population stability.

The study also has practical implications. *P. euphratica* is a dominant constructive species in Tarim River riparian forests and has major ecological significance for sand fixation, habitat maintenance, riparian stability and oasis protection [1,2,13]. The studied populations were not subjected to experimental management or selection; however, one plot was located in an area with planting disturbance, and this was treated as part of the local habitat context rather than as a replicated treatment. Monitoring programs should therefore consider both intermediate-sized individuals and local neighborhood density rather than relying only on seedling abundance as an indicator of recovery.

### 3.4. Limitations and Future Directions

Several limitations of this study should be acknowledged. First, population dynamics were inferred from diameter-class structure rather than from repeated long-term censuses. Therefore, the dynamic indices used here represent structural evidence of demographic transitions, not direct measurements of individual growth, recruitment and mortality. Future studies should use repeated plot censuses to quantify actual transition probabilities among diameter classes.

Second, groundwater depth, soil moisture and soil salinity were not directly incorporated into the statistical models [16], although these factors are likely to interact with neighborhood competition in regulating growth and survival. Future work should combine competition analysis with hydrological and soil measurements to clarify how environmental stress modifies competitive effects.

Third, the present study used three representative 100 m × 100 m plots. This design was suitable for describing local variation within the reserve, but it was not intended to provide a full landscape-level estimate of population behavior. To better represent the species across heterogeneous riparian habitats, future studies should expand the sampling design to include a larger number of plots distributed along distance-to-water, groundwater-depth and disturbance gradients. Such a design would allow for stronger inference about habitat effects and population-level generality.

Finally, the present study focused on intraspecific competition within *P. euphratica* populations. Interactions with shrubs, herbs and other co-occurring woody species may also influence recruitment, growth and survival. Despite these limitations, the integration of diameter-class structure, dynamic indices and distance-dependent competition provides a useful structural framework for identifying demographic bottlenecks and competition-sensitive stages in long-lived arid riparian trees.

## 4. Materials and Methods

### 4.1. Study Area

The study was conducted in the Tarim *Populus euphratica* National Nature Reserve, located in the Tarim River Basin of the Xinjiang Uygur Autonomous Region, northwestern China. The reserve covers approximately 395,420 ha and represents one of the largest and most intact natural *Populus euphratica* forests in the world. It plays a critical role in maintaining ecological security in arid inland river systems.

The region is characterized by a typical temperate continental arid climate [31,38]. Annual precipitation is generally less than 50 mm, whereas potential evaporation exceeds 2000 mm. Mean annual temperature ranges from 9.7 to 10.8 °C, with extremely high summer temperatures and large diurnal temperature fluctuations. Vegetation growth is highly dependent on groundwater and seasonal river flooding due to the extremely limited precipitation [8,25,26,39]. The dominant tree species is *Populus euphratica*, accompanied by *Tamarix* spp., *Phragmites australis*, and other desert shrubs and herbaceous species.

**Figure 7:**
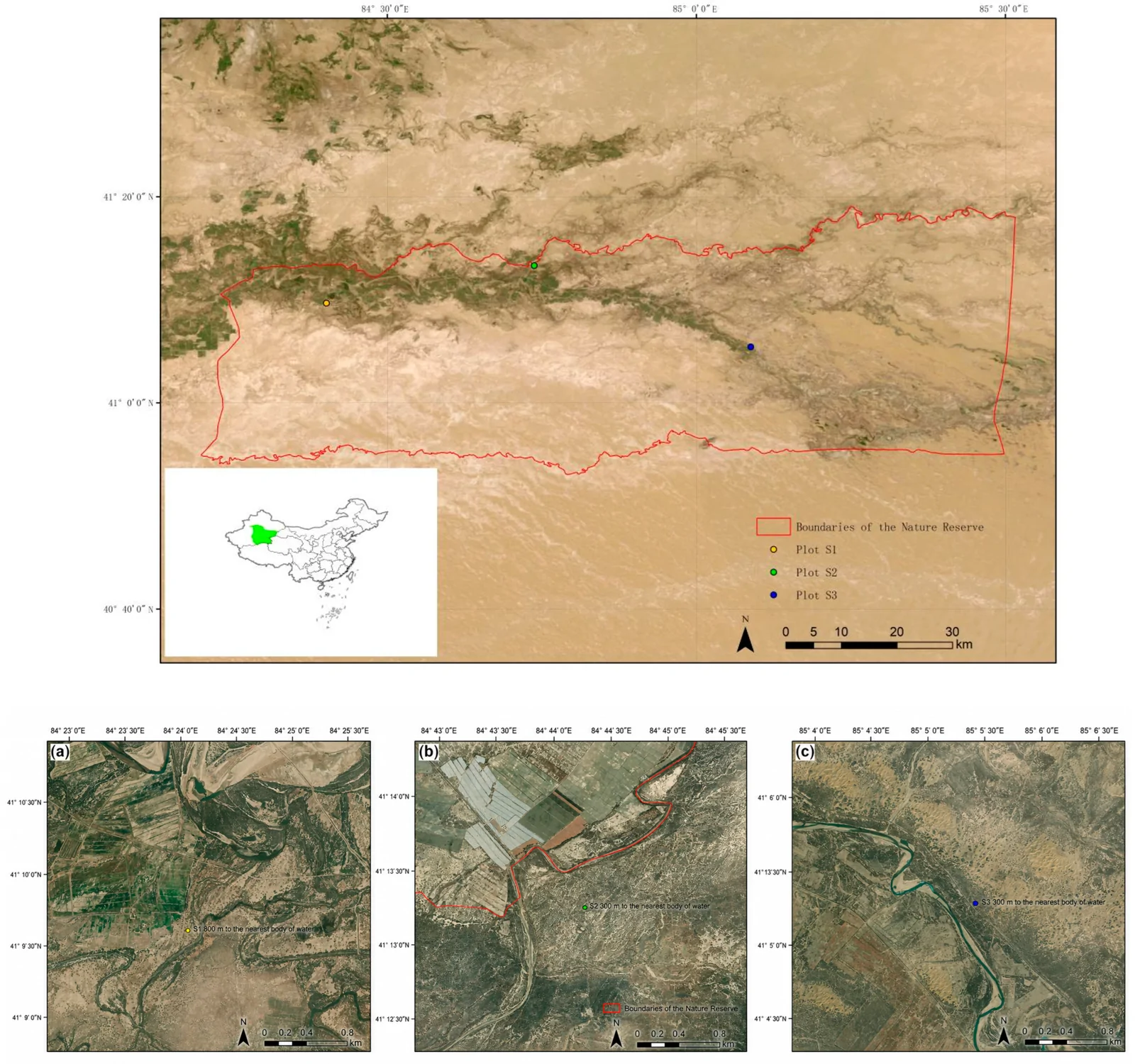
Study area and sampling plots in the Tarim River Basin. Enlarged satellite-map views showing the local landscape context of the three sampling plots: (**a**) S1, (**b**) S2 and (**c**) S3. Colored points indicate the centers of the sampling plots, and labels show the straight-line distance from each plot to the nearest body of water. The red line in panel (**b**) indicates the boundary of the Tarim *Populus euphratica* National Nature Reserve. Scale bars and north arrows are shown in each panel. Base imagery source: ArcGIS World Imagery (ArcGIS 10.7).

The study area exhibits substantial environmental heterogeneity in terms of groundwater depth, soil moisture, salinity, and flood disturbance intensity. Such heterogeneity provides favorable conditions for investigating population structure, demographic dynamics and competitive interactions within *P. euphratica* populations (Figure 7 and Table 6). In Figure 7b, the distances shown for S1–S3 indicate straight-line distances from plot centers to the nearest body of water.

### 4.2. Field Survey and Data Collection

Field investigations were conducted from 2 July to 15 July 2025. Three representative sample plots (S1, S2, and S3) were established within the reserve to represent different habitat conditions and population development stages. The location of each plot was selected to ensure relatively homogeneous topography and vegetation composition while maintaining independence among plots.

The three plots were not intended to represent replicated experimental treatments but to capture major local variations in population structure and habitat context within the reserve. Therefore, plot-level comparisons were used to describe spatial heterogeneity rather than to infer treatment effects. All individual-level competition analyses were conducted using measured tree attributes and neighborhood density.

Within each plot, all *Populus euphratica* individuals were surveyed and measured. For each individual tree, diameter at breast height (DBH, cm), tree height (m), crown width along the x-axis (m), crown width along the y-axis (m), and spatial coordinates (X and Y) were recorded. Crown area (m^2^) was calculated using the projected crown dimensions.

The local neighborhood density of each individual was quantified as the number of neighborhood trees within a 10 m radius. This variable was used as an indicator of local crowding and competition pressure. The final dataset included the following variables:

- Plot identification (S1, S2, S3).
- Diameter at breast height (DBH).
- Tree height.
- Crown width (x-direction).
- Crown width (y-direction).
- Crown area.
- Hegyi competition index.
- Spatial coordinates (X and Y).
- Local neighborhood density within a 10 m radius.

These variables were subsequently used for analyses of population structure, population dynamics, and competition intensity.

### 4.3. Diameter-Class Classification

Population structure was evaluated using diameter-class distributions. Diameter at breast height was used as a surrogate measure of age because direct age determination of *P. euphratica* individuals is difficult under natural field conditions [15,20].

Based on previous research, all individuals were classified into 12 diameter classes at 4 cm intervals: Class I, DBH < 4 cm; Class II, 4 ≤ DBH < 8 cm; Class III, 8 ≤ DBH < 12 cm; …; and Class XII, DBH ≥ 44 cm.

The number and proportion of individuals in each diameter class were calculated for each plot and for the entire population. Diameter-class distributions were used to assess population regeneration status and infer developmental trends. According to classical population ecological theory, inverse J-shaped distributions indicate growing populations with active regeneration, whereas unimodal or declining distributions may indicate limited recruitment and potential population degradation.

Comparisons among plots were conducted to evaluate spatial variation in population structure and regeneration capacity.

### 4.4. Population Dynamics Indices

Population dynamics were assessed using dynamic indices calculated between adjacent diameter classes [15,24]. These indices quantify transitions among size classes and provide information on recruitment, growth, and mortality processes.

The dynamic indices were calculated as follows:

(1) $$V_{n}=\frac{S_{n}-S_{n+1}}{\max(S_{n},S_{n+1})}\times 100\%,$$

(2) $$V_{pi}=\frac{\sum_{n=1}^{k-1}S_{n}V_{n}}{\sum_{n=1}^{k-1}S_{n}},$$

(3) $$V_{pi}^{\prime }=\frac{\sum_{n=1}^{k-1}(S_{n}V_{n})}{\min(S_{1},S_{2},\ldots ,S_{k})\times \sum_{n=1}^{k-1}S_{n}},$$

(4) $$P_{\max}=\frac{1}{k\min(S_{1},S_{2},\ldots ,S_{k})},$$

where S_n_ and S_n+1_ are the numbers of individuals in the n-th and (n + 1)-th diameter classes, respectively; k is the total number of diameter classes; V_n_ is the dynamic index between adjacent diameter classes; V_pi_ is the overall population dynamic index; V_pi_′ is the corrected population dynamic index; P_max_ is the maximum dynamic index; and the prime symbol (′) denotes the corrected form of V_pi_.

To reduce the influence of unequal class frequencies, the corrected dynamic index (V_pi_′) was further calculated according to established methods.

Population stability was evaluated using the maximum dynamic index (P_max_), which reflects the overall developmental tendency of the population under natural conditions.

Positive values of V_pi_ indicate increasing abundance between adjacent diameter classes, whereas negative values indicate decreasing abundance. Larger absolute values represent stronger demographic changes.

Dynamic indices were calculated separately for each plot and for the overall population to compare population developmental trajectories among different habitat conditions.

### 4.5. Competition Index

Intraspecific competition was quantified using the Hegyi competition index, one of the most widely used distance-dependent competition indices in forest ecology [30].

In this study, to minimize the impact of the edge effect in the plots, the target trees were *P. euphratica* located within a 94 m × 94 m area at the center of each plot, and non-tillering *P. euphratica* within a 6 m radius of these trees were designated as competitor trees.

The competition index for each target tree was calculated as follows:

(5) $$CI=\sum_{j=1}^{n}\left(\frac{D_{j}}{D_{i}}\right)\frac{1}{L_{ij}},$$

where *CI* represents the competition index; *D_i_* represents the DBH of the target tree; *D_j_* is the DBH of the competing tree; *L_ij_* represents the distance between the target tree and the competing tree; and n represents the number of competing trees.

Higher values of the Hegyi index indicate stronger competitive pressure exerted on the target individual.

This index incorporates both the size asymmetry and spatial proximity of neighborhood individuals and is therefore particularly suitable for assessing competitive interactions within unevenly aged natural forests.

### 4.6. Statistical Analysis

All statistical analyses were conducted to evaluate relationships between competition intensity and tree structural attributes in *Populus euphratica* populations. The Hegyi competition index was used as the response variable. Diameter at breast height (DBH), tree height, crown area and local neighborhood density were used as explanatory variables. Local neighborhood density was defined as the number of conspecific neighborhood trees within a 10 m radius.

Pearson correlation analysis was first used to examine pairwise relationships among competition intensity and explanatory variables. Simple linear regression models were then fitted with the Hegyi competition index as the dependent variable and DBH, tree height, crown area or local neighborhood density as a single predictor. A multiple linear regression model was further established to evaluate the combined effects of all explanatory variables on competition intensity. Variance inflation factors (VIFs) were calculated to assess multicollinearity, with VIF values below 10 considered acceptable.

Nonlinear models were fitted to describe potential nonlinear responses of competition intensity to tree size and local neighborhood density. Exponential, power-law and log-transformed models were used where appropriate. For nonlinear and log-transformed models, only observations with positive competition index and predictor values were included when required. Model performance was evaluated using R^2^, adjusted R^2^, residual standard error (RSE), parameter significance and residual diagnostics. For nonlinear least-squares models, RSE and parameter significance were used as the main criteria for model comparison.

All analyses and visualizations were performed in R 4.4.2 using the packages readxl, dplyr, tidyr, ggplot2, patchwork and car. Statistical significance was assessed at *p* < 0.05.

## 5. Conclusions

This study integrated diameter-class structure, population dynamic indices and distance-dependent competition analysis to examine *Populus euphratica* population persistence in an arid riparian ecosystem. The population exhibited an inverse J-shaped diameter distribution, indicating active recruitment, but the reduced representation of intermediate and large individuals showed that recruitment alone does not ensure continuous demographic progression.

Dynamic indices revealed stage-dependent population development, with intermediate diameter classes forming a clear demographic bottleneck. Competition intensity was size-dependent and density-dependent, decreasing with increasing tree size but increasing with local neighborhood density. These patterns suggest that intraspecific competition may act as a demographic filter during transitions among developmental stages.

Overall, *P. euphratica* persistence was closely associated with the coupling of recruitment, demographic transitions and neighborhood competition. These findings highlight the importance of considering both demographic bottlenecks and plant–plant interactions when assessing the persistence of dominant tree species in arid riparian ecosystems.

## Figures and Tables

**Table 1:** Dynamic indices among adjacent diameter classes.

Plot	V1	V2	V3	V4	V5	V6	V7	V8	V9	V10	V11
S1	0.62	0.33	0.13	0.42	0.13	−0.13	0.4	−0.18	0.73	−0.25	0
S2	0.73	0.31	0.53	0.43	0.44	0.76	0.75	1	−1	1	−1
S3	0.93	0.41	0.1	0.11	−0.2	0.2	−0.33	−0.2	−0.12	0.06	−0.24
Total	0.76	0.32	0.47	0.41	0.32	0.46	0.26	−0.16	0.19	0.05	−0.31

**Table 2:** Population stability indices.

Plot	V_pi_	V_pi_′	P_max_
S1	0.39	0.02	0.03
S2	0.62	0.03	0.08
S3	0.59	0.03	0.01
Total	0.59	0.00	0.00

**Table 3:** Simple linear regression models.

Predictor	Model	Estimate Direction	R^2^	Adjusted R^2^	*p*-Value
DBH	*CI* = 8.119 − 0.228 *DBH* *	Negative	0.180	0.179	<0.001
Height	*CI* = 11.159 − 0.913 *H* *	Negative	0.167	0.166	<0.001
Crown area	*CI* = 6.607 − 0.157 *CA* *	Negative	0.094	0.093	<0.001
Local neighborhood density	*CI* = 1.357 + 0.177 *D10* *	Positive	0.156	0.155	<0.001

*CI*, *H*, *CA* and *D10* represent competition index, tree height, crown area and local neighborhood density, respectively.

**Table 4:** Multiple linear regression model.

Variable	Estimate	SE	t	*p*-Value	VIF
Intercept	6.868	0.595	11.534	<0.001	—
DBH	−0.098	0.041	−2.388	0.017	7.163
Height	−0.618	0.117	−5.264	<0.001	3.392
Crown area	0.068	0.027	2.493	0.013	3.488
Local neighborhood density	0.138	0.014	9.596	<0.001	1.273

Model statistics: R^2^ = 0.271, adjusted R^2^ = 0.268, F = 83.53, *p* < 0.001. VIF was not calculated for the intercept.

**Table 5:** Nonlinear and log-transformed models.

Predictor	Model Type	Equation	R^2^ *	RSE	*p*
DBH	Exponential	*CI* = 17.15 exp(−0.138 *DBH*)	-	4.930	<0.001
DBH	Power-law	*CI* = 35.22 *DBH*^−0^·^921^	-	4.887	<0.001
Crown area	Log-transformed	ln(*CI*) = 2.912 − 1.022 ln(*CA*)	0.433	0.944	<0.001
Local neighborhood density	Exponential	*CI* = 2.600 exp(0.029 *D10*)	-	5.488	<0.001
Local neighborhood density	Log-transformed	ln(*CI*) = −1.486 + 0.919 ln(*D10*)	0.408	0.958	<0.001

R^2^ was reported only for log-transformed linear models; nonlinear least-squares models were compared using residual standard error.

**Table 6:** Characteristics of the sampling plots.

Plot	Longitude (°E)	Latitude (°N)	Elevation	Distance to the Nearest Body of Water (m)	Disturbance	Plot Size
S1	84.40	41.16	862.7	800	—	100 m × 100 m
S2	84.74	41.22	853.1	300	Planting	100 m × 100 m
S3	85.09	41.09	848.0	300	—	100 m × 100 m

## Data Availability

The data that support the findings of this study are not publicly available because they were generated under a technical service project conducted in the Xinjiang Tarim Poplar National Nature Reserve and are subject to project data management requirements. Data may be made available by the corresponding author upon reasonable request and with permission from the Xinjiang Tarim Poplar National Nature Reserve Administration.

## Supplementary Materials

The following supporting information can be downloaded at: https://www.mdpi.com/article/10.3390/plants15172662/s1, Figure S1. Scatterplot matrix showing pairwise relationships among DBH, tree height, crown area, local neighborhood density and competition index. Lower panels show scatterplots with fitted linear regression lines, diagonal panels show density distributions, and upper panels show Pearson correlation coefficients. Asterisks indicate statistical significance: *** *p* < 0.001.

## Abbreviations

The following abbreviations are used in this manuscript:

DBH	Diameter at breast height
CI	Competition index
VIF	Variance inflation factor
RSE	Residual standard error
V_pi_	Overall population dynamic index
V_pi_′	Corrected population dynamic index
P_max_	Maximum dynamic index
