# Supplementary material for: Demographic Bottlenecks and Size-Dependent Competition Shape Populus euphratica Persistence
Source: Plants (Basel). 2026 Aug 30;15(17):2662. doi: 10.3390/plants15172662 (PMC13567013; doi:10.3390/plants15172662)
Supplement: Supplementary file 1 [file plants-15-02662-s001.zip › Supplementary Figure S1.pdf]

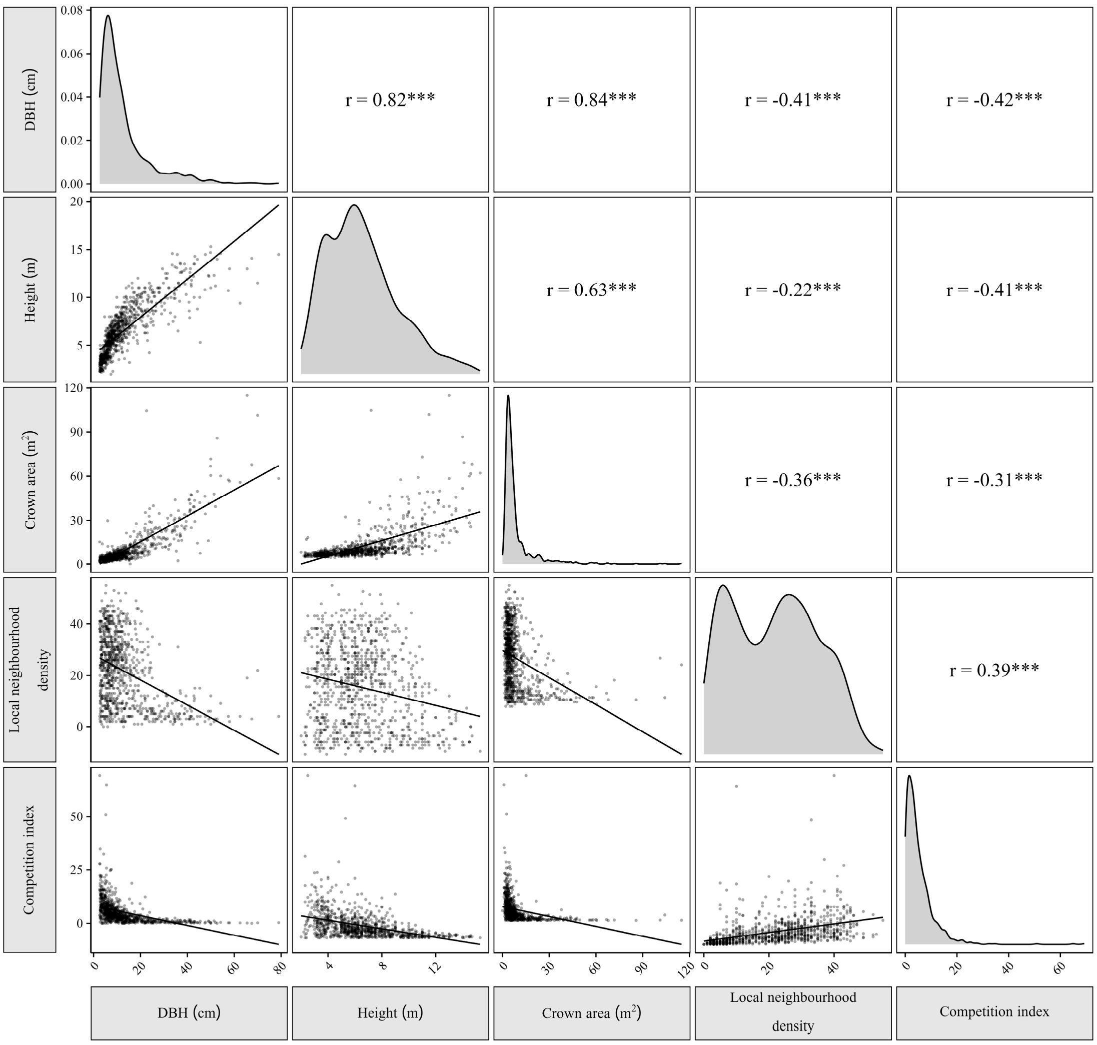

Figure S1. Scatterplot matrix showing pairwise relationships among DBH, tree height, crown area, local neighbourhood density and competition index. Lower panels show scatterplots with fitted linear regression lines, diagonal panels show density distributions, and upper panels show Pearson correlation coefficients. Asterisks indicate statistical significance: * P < 0.05, ** P < 0.01 and *** P < 0.001.
